# Long-term outcomes of autologous cell therapy in patients with ischaemic diabetic foot ulcers and no-option chronic limb threatening ischaemia

**DOI:** 10.1007/s00592-026-02721-5

**Published:** 2026-06-22

**Authors:** Meloni Marco, Bellizzi Ermanno, Uccioli Luigi, Morosetti Daniele, Giurato Laura, Bonanni Federico Rolando, Ruotolo Valeria, Andreadi Aikaterini, Bellia Alfonso, Lauro Davide

**Affiliations:** 1 Department of Systems Medicine, University of Rome “Tor Vergata”, Rome, 00133 Italy; 2 Division of Endocrinology and Diabetology, Department of Medical Sciences, Fondazione Policlinico “Tor Vergata”, Rome, 00133 Italy; 3 UOC Diabetic Foot, IRCCS MULTIMEDICA, Sesto San Giovanni, Milan, Italy; 4 Division of Endocrinology and Diabetes, CTO Andrea Alesini Hospital, Rome, 00145 Italy; 5 Interventional Radiology Unit, Dipartimento Processi Assistenziali Integrati, University of Rome Tor Vergata, Rome, Italy

**Keywords:** Diabetes, Diabetic foot, Peripheral arterial disease, Peripheral blood mononuclear cells, Revascularization

## Abstract

**Aim:**

Patients with ischaemic diabetic foot ulcers (DFUs) and no-option chronic limb-threatening ischaemia (CLTI) are reported to have a high risk of major amputation and mortality. Peripheral blood mononuclear cell (PB-MNCs) therapy has emerged as a promising adjunctive strategy, but long-term data remain limited. The study aimed to evaluate the long-term outcomes of PB-MNC therapy in patients with DFUs and NO-CLTI.

**Methods:**

This prospective, single-centre, observational, controlled study included patients with ischaemic DFUs and no-option CLTI enrolled between January 2017 and September 2023. No-option CLTI was defined by a technical failure of lower limb revascularization with the absence of any below-the-ankle vessel and/or indirect revascularization with TcPO₂ values < 30 mmHg in the wound angiosome area. Patients were divided into two groups: those receiving PB-MNCs therapy plus standard of care (SoC) and those treated only with SOC based on conventional guidelines. PB-MNCs were administered by intramuscular injections along the anatomical area of the wound-related artery (2 or 3 cycles, 21–42 days apart). At 24 months of follow-up, outcomes assessed were healing, major amputation, and mortality. In the PB-MNC group, hospital readmission for recurrent CLTI requiring repeat revascularization was also recorded.

**Results:**

Overall, 78 patients were included: 48 in the PB-MNCs group and 30 in the conventional therapy group. The mean age was 73.7 years, 84.6% were male, and most had type 2 diabetes (92.3%). At the baseline assessment, 71.8% of DFUs were infected, 83.3% were > 5 cm², and 88.5% had gangrene. At 24 months, the PB-MNCs group showed a higher rate of healing (60.4 vs. 20%, *p* < 0.0001), lower rate of major amputation (12.5 vs. 26.7%, *p* = 0.0004), and mortality (27.1 vs. 46.7%, *p* = 0.0002) than the control group. CLTI recurrence requiring readmission occurred in 3/48 (6.2%) among patients in the PB-MNC group.

**Conclusions:**

In patients with ischaemic DFUs and NO-CLTI, adjunctive PB-MNCs therapy was associated with better long-term outcomes in comparison to conventional therapy.

## Introduction

 Diabetic foot disease (DFD) represents one of the most severe complications of diabetes, imposing a substantial clinical, social, and economic burden on both patients and healthcare systems [1]. It arises from the interplay between peripheral neuropathy and peripheral arterial disease (PAD), with diabetic foot ulceration (DFU) being its most advanced and clinically relevant presentation.

In recent years, an increasing prevalence of ischaemic DFUs compared with neuropathic lesions has been reported [2–4]. The coexistence of PAD significantly worsens prognosis, and is strongly associated with delayed wound healing, higher rates of major amputation, and increased mortality [2, 3].

Restoration of adequate blood flow is a key therapeutic goal in patients with ischaemic DFUs. For this reason, lower limb revascularization is frequently required to enhance tissue perfusion and support ulcer healing [5]. Current strategies aim to re-establish direct blood flow below the ankle (BTA), ideally by targeting the artery feeding the specific angiosome where the ulcer is located. The concept of the wound angiosome refers to the anatomical territory supplied by a main foot artery—such as the pedal, medial plantar, or lateral plantar artery—and its collateral branches [6].

Despite being widely performed in specialised vascular centres with high procedural success rates, revascularization fails in up to one-quarter of patients [7]. These failures are mainly due to the inability to effectively treat complex arterial stenosis or occlusions, resulting in persistent ischaemia either diffusely in the foot or within the affected angiosome [7]. Importantly, unsuccessful revascularization is a strong independent predictor of major amputation [7–11]. Patients in whom no effective revascularization can be achieved are classified as having no-option chronic limb-threatening ischaemia (CLTI), a condition associated with a higher risk of major amputation and mortality [7].

In this challenging clinical setting, autologous cell therapy (ACT), and in particular the use of peripheral blood mononuclear cells (PB-MNCs), has emerged as a promising therapeutic approach. Evidence suggests that PB-MNC therapy may improve tissue perfusion, promote wound healing, and increase limb salvage rates in diabetic patients with no-option CLTI [12, 13]. Consequently, in some multidisciplinary diabetic foot units, this strategy is increasingly adopted as an adjunctive treatment in patients who are not candidates for, or who have failed, conventional revascularization.

However, although short- and mid-term outcomes appear encouraging, robust data on the long-term efficacy of PB-MNC therapy—especially regarding limb preservation, survival, and recurrence of limb ischaemia requiring hospital readmission—remain limited.

On this basis, the present study was designed to investigate the long-term outcomes of PB-MNC therapy in patients with ischaemic DFUs and no-option CLTI.

## Methods

### Patient’ selection

The study was designed as a prospective, single-centre, controlled study involving a cohort of patients with ischaemic or neuro-ischaemic diabetic foot ulcers (DFUs). Participants were enrolled between January 2017 and September 2023 and had previously undergone lower limb revascularization.

Eligible patients were those presenting with ischaemic or neuro-ischaemic DFUs classified as stages 1C, 2C, 3C, and 1D, 2D, 3D according to the University of Texas Classification [14], in whom revascularization had been technically unsuccessful. Technical failure was defined as the inability to cross stenotic or occluded vessels and consequently restore adequate foot perfusion. Accordingly, the study included patients with “desert foot” (i.e., absence of below-the-ankle vessels such as pedal and plantar arteries) and/or those in whom direct revascularization of the wound-related artery had failed, resulting in persistent ischaemia within the wound angiosome, as indicated by TcPO₂ values below 30 mmHg [15]. Table 1.

**Table 1:** Inclusion and exclusion criteria for the study

Inclusion criteria	Exclusion criteria
Presence of diabetes Presence of foot ulcer; Ischaemic/neuro-ischaemic DFU stage 1 C-D, 2 C-D, 3 C-D according to University of Texas Classification; Unsuccessful below-the-ankle revascularization: desert foot and/or an absence of direct flow to the wound angiosome area with TcPO2 values < 30 mmHg; Presence of at least one below-the-knee vessel (anterior tibial artery, posterior tibial artery, peroneal artery);	Absence of diabetes; Absence of foot ulcer; Successful revascularization below-the-ankle with TcPO2 > 30 mmHg in the wound angiosome area; Indication for primary amputation due to absence of anatomical suitability for surgical foot salvage; Reduced life expectancy (less than 6 months); Immunosuppressive therapy; Active neoplastic disease under therapy; Severe cognitive impairment; Lost to follow-up; Follow-up shorter than 2 years;

*DFU* diabetic foot ulcer, *TcPO2* transcutaneous pressure of oxygen

Patients were excluded if they had an indication for primary amputation due to the lack of anatomical feasibility for limb salvage through any surgical intervention, absence of patent below-the-knee vessels, limited life expectancy, ongoing immunosuppressive treatment, active malignancy, or severe cognitive impairment. Table 1.

Among the whole population, two groups were identified: a group treated with PB-MNCs therapy (study group) in addition to standard care and a historical group treated with conventional therapy (control group) which according to the International Working Group on the Diabetic Foot (IWGDF) guidelines means they received the best medical therapy [16].

The conventional group included the historical group of no-option CLTI treated between 2017 and 2019 in the period in which the cell therapy system was not available, while those treated with PB-MNC therapy were enrolled between 2020 and September 2023 when the cell therapy system was available. Patients included in the study group were those who had received at least 2 cycles of PB-MNC therapy during their enrolment.

All included patients were managed at the Unit of Endocrinology and Metabolic Diseases, a tertiary-level diabetic foot care centre at the University Hospital of Tor Vergata in Rome, Italy.

Patient care was delivered by a multidisciplinary diabetic foot team (MDFT) according to a standardized limb salvage protocol, in line with the recommendations of the IWGDF [16]. Global management included vascular and infection control, offloading strategies, surgical interventions tailored to individual clinical conditions, and optimization of diabetes, its complications, and associated comorbidities.

The MDFT consisted of diabetologists, endocrinologists, interventional radiologists, vascular surgeons, podiatrists, and specialized nurses. Diabetologists served as team leaders, overseeing both medical and surgical management in accordance with the diabetic patient centred model [17]. Those with specific expertise in peripheral vascular disease were also responsible for performing the cell therapy procedures.

Notwithstanding that the study and control group were treated in two non-contemporaneous historical periods that can potentially interfere withthe measure of outcomes, each group were managed by a similar local protocol in accordance with international guidelines using the same approach in terms of revascularization procedure, management of infection and medical therapy (glucose lowering-therapy, cardiovascular drugs) although the use of more recent glucose lowering-therapy (iSGLT2 and GLP1-RAs) has been implemented in the last year. In addition, both groups were managed by the same multidisciplinary team including surgical, medical and vascular teams.

At baseline, demographic data, clinical variables, and wound characteristics were systematically recorded and compared between groups (study group managed by adjunctive PB-MNCs therapy and control group managed by conventional therapy).

### Clinical features and wound assessment

Among comorbidities, hypertension, hypercholesterolemia, ischaemic heart disease (IHD), heart failure (HF), cerebrovascular disease (CVD), and end-stage renal disease (ESRD) were recorded at baseline. Smoker status was considered only in the case of active smokers at the time of the enrolment. On assessment, all patients received the best medical therapy according to guideline recommendations, including antiplatelet therapy (acetylsalicylic acid or clopidogrel), a low dose of rivaroxaban, statins, Glucagon-like peptide 1 receptor agonists (GLP-1RAs) and/or sodium-glucose cotransporter-2 inhibitors (iSGLT-2) according to each specific case [5, 18].

Based on the IWGDF definitions [19], the following wound characteristics were recorded: size, depth, infection, osteomyelitis, presence of gangrene. Infection was diagnosed in relation to the presence of clinical signs such as swelling, induration, redness, warmth, tenderness, purulent discharge, pain and/or the presence of systemic signs including fever, fatigue, sepsis [20]. Bone infection in cases of bone involvement and/or bone exposure in association with radiological signs documented by x-rays or magnetic resonance imaging (MRI) if required [20].

### Cell therapy procedure

All patients enrolled in the study underwent adjunctive cell therapy with a concentrated preparation of autologous peripheral blood mononuclear cells (PB-MNCs). The treatment consisted of targeted injections of PB-MNCs into the affected foot, specifically along the course of the occluded artery responsible for perfusing the ischaemic wound angiosome (see Table 2).

**Table 2:** Description of foot angiosome and the targeted artery for cell injection according to the wound location

Foot angiosome and anatomical vascular site of cell injection	Wound location
Posterior tibial artery	
(1) Posterior medial malleolar artery and posterior medial calcaneal artery	Medial-plantar surface of the heel
(2) Medial plantar artery	Medial column and medial-plantar surface of the midfoot and forefoot, plantar surface of the hallux and the medial-plantar surface of the 2nd toe
(3) Lateral plantar artery	Lateral column and lateral-plantar surface of the midfoot and forefoot, lateral plantar surface of the 2nd toe, the plantar surface of the 3rd and 4th toe and the plantar and lateral surface of the 5th toe
Anterior tibial artery	
(1) Pedal artery and its branches (arcuate artery and intermetatarsal arteries)	Dorsum of the foot, dorsal surface of toes, dorsal peri-malleolar area
Peroneal artery	
(1) Distal part (below-the-ankle) of peroneal artery	Lateral and plantar surface of the heel
(2) Lateral calcaneal branch of the peroneal artery	Lateral surface of the rearfoot extended until the proximal 5th metatarsal and superiorly until to the lateral malleolus

PB-MNCs were isolated using the HemaTrate^®^ Blood Filtration System (Cook Regentec, Indianapolis, IN, USA). Cell concentration was achieved through a gravity-based filtration method [21]. Briefly, 120 mL of peripheral blood anticoagulated with acid-citrate-dextrose (ACD) was introduced into the upper chamber of the system, allowing for gravitational separation. The concentrated mononuclear cell fraction was subsequently collected in a sterile saline backflush, making it immediately available for administration. All procedures were carried out in a dedicated foot surgery setting.

The PB-MNCs suspension was administered via intramuscular injections into the foot (below the ankle), following the anatomical distribution of the wound-related artery and its collateral branches. Injections of 1 mL per site were performed at intervals of 1–1.5 cm, reaching a depth of approximately 1–2 cm, using a 27-gauge needle. In cases where patients experienced pain during the procedure, local below-the-ankle anaesthesia with 2% lidocaine (10–20 mL) was provided.

The treatment was repeated two or three times overall, 21–42 days apart. The evaluation of wound angiosome perfusion before starting PB-MNCs therapy and after the last procedure (usually 2–4 weeks later) was performed through the measurement of skin perfusion using transcutaneous oxygen pressure (TcPO2).

From 2 to 4 weeks after the last PB-MNCs procedure, all patients received surgical treatment (toe amputation, ray amputation, sequestrectomy, bone resection, trans-metatarsal amputation, etc.) based on each clinical presentation and TcPO2 values, otherwise the local wound management with healing by secondary intention was adopted according to the characteristics of the target ulcer.

In the case of persistence of foot/wound ischaemia, persistent or increased foot pain, significant extension of gangrene, concomitant infection not suitable for conservative therapy, major amputation was performed.

In patients belonging to PB-MNCs group, except for deceased and amputees, TcPO2 was repeated after 2 years of follow-up and compared with the baseline values.

The study was performed according to the Declaration of Helsinki and was approved by the Ethics Committee (protocol number MC-01-2016; 151/16).

### Outcome measures

The primary endpoint was to evaluate amputation-free survival at 24 months. The secondary outcomes were healing in surviving patients, major amputation, and survival. All outcome measures were recorded after 2 years of follow-up. Wound healing was defined as complete epithelialization of the target wound and maintenance of the healed epithelialized surface for at least 2 weeks. Healing was defined as complete wound healing without major amputation, including both patients who required a minor amputation to achieve wound healing and those who recovered without a minor amputation. The rate of minor amputation was also reported separately. Minor amputation was considered any amputation below the ankle, while major amputation was considered any amputation above the ankle. Outcomes were recorded for both groups and compared.

In the group treated with PB-MNCs therapy, the rate of hospital re-admission for a new lower limb revascularization in the affected limb due to CLTI recurrence was recorded. CLTI recurrence was considered in thecase of non-healing DFU (absence of granulation tissue, absence of epithelialization signs, and absence of reduction of ulcer size during the follow-up excluding other clinical conditions), recurrence of pain, and/or ulcer relapse in association with clinical signs of foot ischaemia, TcPO2 values < 30 mmHg in the wound angiosome area and documented stenosis/obstruction at colour ultrasound duplex.

### Statistical analysis

Statistical analysis was performed using SAS software (JMP12; SAS Institute, Cary, NC). Data were expressed as means ± SD. Comparisons between groups were reported by the chi-square test (frequency data) or Student’s t-test (continuous data). Univariate logistic regression analysis was performed for all potential predictive variables (age, sex, diabetes duration, hypertension, dyslipidaemia, being an active smoker, IHD, ESRD on dialysis, heart failure, ulcer size, infection, heel location, multiple ulcers/gangrene), and odds ratios (ORs) with 95% CIs were given to measure the association with outcomes of interest. Then, all potential predictors were entered simultaneously in a multivariate logistic regression model. These models yielded a set of variables that best predicted the outcome. For all analyses, a p-value < 0.05 was considered statistically significant. Ultimately, results are unadjusted and exploratory.

## Results

**Fig. 1:**
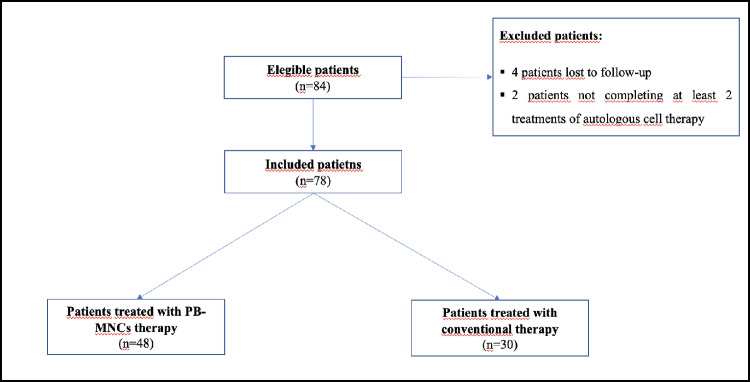
Flow diagram of the study. PB-MNCs: Peripheral Blood-Mononuclear cells

Among 84 patients retrospectively eligible for the study, 4 patients were excluded due to lost follow-up and 2 for not receiving at least 2 cycles of PB-MNCs therapy. Overall, 78 patients with no-option CLTI meeting the inclusion criteria were included, 48 treated with PB-MNC therapy and 30 with conventional therapy. Figure 1 In the cohort of the PB-MNCs group, 40 patients received 3 cycles of PB-MNCs and 8 patients received 2 cycles.

Overall, included patients had a mean age of 73.7 years, the majority were male (84.6%) and almost all of them were affected by type 2 diabetes (T2D) with a long diabetes duration (> 20 years). A high rate of concomitant cardiovascular co-morbidities was found at the assessment, mainly IHD (75.6%) and heart failure (44.9%). Table 3.

**Table 3:** Baseline characteristics on assessment of the total population

Variable	Whole population (*N* = 78)	PB-MNCs group (*N* = 48)	Conventional therapy group *N* = 30	*P* value
Age (years)	73.7 ± 9.1	76.6 ± 8.4	69 ± 10.3	0.06
Sex (male) n (%)	66 (84.6)	43 (89.6)	23 (76.7)	0.07
Diabetes (type 2) n (%)	72 (92.3)	47 (97.9)	25 (83.3)	0.04
Diabetes duration (years)	23 ± 9.4	23.6 ± 7.1	22.1 ± 13	0.1
HbA1c mmol/mol (%)	66 ± 17 (8.2 ± 3.7)	68 ± 13 (8.3 ± 3.3)	64 ± 23 (8 ± 4.3)	0.6
Dyslipidaemia n (%)	56 (71.8)	35 (72.9)	21 (75)	0.2
Hypertension n (%)	67 (85.9)	45 (93.7)	22 (73.3)	0.002
ESRD n (%)	11 (14.1)	3 (6.2%)	8 (26.7)	0.0001
IHD n (%)	59 (75.6)	35 (72.9)	24 (80)	0.08
Heart failure n (%)	35 (44.9)	16 (33.3)	19 (63.3)	0.0001
CVD n (%)	21 (6.9)	14 (29.1)	7 (23.2)	0.4
Active Smoker	8 (10.3)	5 (10.4)	3 (10)	1
HB (gr/dl)	10.4 ± 2.2	10.8 ± 2.4	9.9 ± 1.8	0.3
Ulcer size (> 5 cm^2^) n (%)	65 (83.3)	44 (91.7)	21 (70)	0.0002
Infection n (%)	56 (71.8)	37 (77.1)	19 (63.3)	0.06
Osteomyelitis n (%)	37 (47.4)	20 (41.7)	17 (56.7)	0.07
Gangrene n (%)	69 (88.5)	45 (93.7)	24 (80)	0.07
Heel location n (%)	21 (26.9)	10 (20.8)	11 (36.7)	0.04
TcPO2 baseline (mmHg)*	16 ± 8	17 ± 10	14 ± 5	0.09

*HbA1c* glycated haemoglobin, *ESRD* end-stage renal disease, *IHD* ischaemic heart disease, *CVD* cerebrovascular disease, *Hb* haemoglobin, *TcPO2* transcutaneous pressure of oxygen

*TcPo2 was evaluated after failed revascularization procedure

Regarding DFU’ characteristics, on assessment the majority were infected (71.8%), larger than 5 cm^2^ (83.3%) and nearly all of them presented with gangrene (88.5%); 20.8% of patients showed a wound located in the heel (26.9%). The mean TcPO2 values at the assessment (after failed revascularization procedures) was 16 mmHg. Table 3.

The two groups, PB-MNCs and conventional therapy, differed at baseline for some clinical and wound characteristics. The conventional therapy group had higher rates of heart failure and ESRD, and a lower rate of hypertension; the PB-MNCs therapy group had a higher rate of ulcer size >5cm^2^ and infection, and a lower rate of ulcers located on the heel, even if infection and wound location were not statistically significant (Fig. 1).

**Fig. 2:**
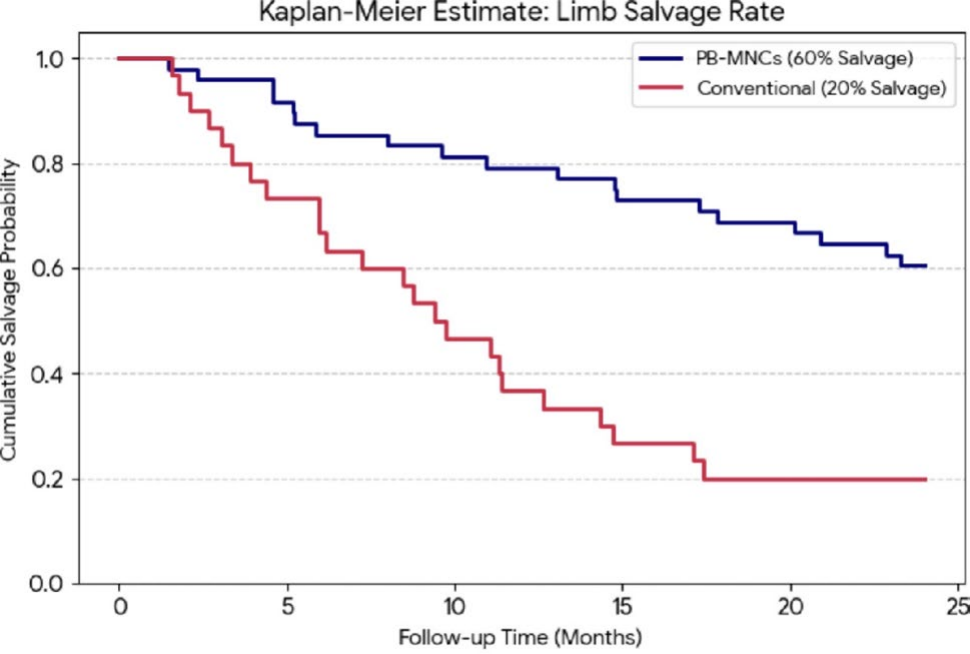
Amputation-free survival by Kaplan-Meier curve

The 2-year amputation-free survival was 47.4% in the whole population, and 60.4% vs. 26.7% (*p* < 0.0001) in the PB-MNCs group and conventional therapy group respectively. The PB-MNCs group demonstrated a significantly higher rate of healing (60.4 vs. 20%, *p* < 0.0001), lower rate of major amputation (12.5 vs. 26.7%, *p* = 0.0004) and mortality (27.1 vs. 46.7%, *p* = 0.0002). Table 4; Fig. 2.

**Table 4:** Outcomes of the total population, study group and control group

Outcomes	Whole population (*N* = 78)	PB-MNCs group (*N* = 48)	Conventional therapy group *N* = 30	*P* value
Amputation-free survival n (%)	37 (47.4)	29 (60.4)	8 (26.7)	< 0.0001
Total healing n (%)	35 (44.9)	29 (60.4)	6 (20)	< 0.0001
Healing without minor amputation	5 (6.4)	4 (8.3)	1 (3.3)	< 0.06
Healing with minor amputation	30 (38.5)	25 (52.1)	5 (16.7)	< 0.0001
Amputee n (%)	14 (17.9)	6 (12.5)	8 (26.7)	0.0004
Deceased n (%)	27 (34.6)	13 (27.1%)	14 (46.7)	0.0002

PB-MNCs group showed a higher rate of healing after minor amputation; however, data are not logically comparable, as several patients in the conventional therapy group had major amputation, while minor amputation was often required in patients with gangrene, such as a demolitive surgical intervention for saving the foot. Table 4.

Among patient deaths (*n* = 27), 20 were related to sudden death due to myocardial infarction, 4 to acute heart failure and additional complications, 2 to infections located in some other district, and 1 to the onset of neoplastic disease. Among deaths in the PB-MNCs group (13), 10 were related to sudden death due to myocardial infarction, 2 to acute heart failure and 1 to some other infection.

The healing mean time in the whole population was 10.7 ± 2.7, while the mean time to major amputation was 4.9 ± 2 months. The mean time to healing in the PB-MNCs group and conventional therapy group was 9.6 ± 2.6 months and 15.8 ± 3.1 months (*p* < 0.0001) respectively, while the mean time to major amputation was 6.7 ± 2.1 months and 3.5 ± 1.9 months (*p* = 0.0001) respectively.

At the multivariate analysis of all potential predictors of outcome found at the univariate analysis, the use of PB-MNCs therapy was a potential predictor of healing and limb salvage, while dialysis, heel location, and multiple ulcers were independent predictors of non-healing and major amputation. Heart disease, including heart failure and ischaemic heart, disease were independent predictors of mortality. Table 5.

**Table 5:** Predictive factors of outcomes of interest at the multivariate analysis

Variables	Healing			Major amputation			Mortality		
	OR	95% CI	*p* value	OR	95% CI	*p* value	OR	95% CI	*p* value
ESRD	0.42	0.23–0.88	0.002	0.32	0.15–0.86	0.0001			
Age (per 10 years increase)							2.2	0.9–3.6	0.08
Heart failure							7.4	4.1–24.6	<0.0001
Ischaemic heart disease							5.2	2.6–18.5.	<0.0001
Autologous cell therapy	3.1	1.7–11.6	0.002	0.07	0.04–0.63	<0.0001			
Heel location	0.09	0.02–0.86	0.0001	2.2	1.9–9.6	0.003			
Ulcer size (> 5 cm^2^)	1.5	0.2–1.8	0.7						
Multiple ulcers	0.5	0.12–0.62	0.0001	5.4	1.4–17.5	0.0001			

Overall, among the PB-MNCs therapy group, 3/48 (6.2%) patients required hospital readmission for the clinical recurrence of CLTI requiring lower limb revascularization. In all cases, a new revascularization was required for the absence of wound healing associated with a local pain and recurrence of stenosis/obstruction in previously treated vessels above-the-ankle, as cell therapy was unable to ensure adequate perfusion of the wound angiosome.

At the end of 2 years of follow-up, patients belonging to the PB-MNCs therapy group, except those who died or underwent amputation, repeated TcPO2 measurement; finally, 29/48 patients underwent TcPO2 evaluation with mean values of 36 ± 8 mmHg, significantly higher than values recorded at baseline (17 ± 10 mmHg), *p* = 0.003. These data come from an exploratory, non-controlled observation.

## Discussion

Data from the present study showed that PB-MNC therapy is associated with potential positive outcomes including long-term benefits, in comparison to no-option CLTI patients treated with conventional therapy. Overall, patients managed by PB-MNCs therapy reported a significantly higher rate of amputation-free survival compared to conventional therapy alone, 60.4% vs. 26.7% (*p* < 0.0001) respectively. Concerning the outcomes of interest, 60.4% of PB-MNCs patients achieved complete wound healing and 12.5% required a major amputation, while those managed with conventional therapy showed only 20% of wound healing and 46.7% of major amputation. At the same time, the mean healing time in the PB-MNCs group was significantly lower than the conventional therapy group (9.6 ± 2.6 months vs. 15.8 ± 3.1 months, *p* < 0.0001), and the mean major amputation time was longer (6.7 ± 2.1 months and 3.5 ± 1.9 months, *p* = 0.0001).

The multivariate analysis confirmed that PB-MNCs therapy was independently related to a higher likelihood of healing and limb salvage, despite not being the only independent predictor of outcomes of interest. In addition, ESRD, heel location and the presence of multiple ulcers were documented to be associated with reduced chances of healing and a higher risk of major amputation.

These findings are consistent with 1-year outcomes reported in the literature for no-option CLTI patients treated with cell therapy. Our research group, in two separate studies, reported healing rates of up to 72% and major amputation rates between 12.5% and 16.4% at 1 year follow-up [22, 23].

Similarly, Ragghianti and Panunzi reported major amputation rates of 20% and 18%, respectively, in no-option CLTI patients with ischaemic DFUs, who were not candidates for conventional revascularization or had failed previous revascularization procedures [24, 25]. To the best of our knowledge, the only study reporting 2-year follow-up data on PB-MNC therapy is the study conducted by Scatena et al. [26], who observed a 10.5% major amputation rate and a wound healing rate exceeding 80%. Overall, our findings support the long-term effectiveness described in that study. Also, in the mentioned study, the limb salvage achieved with autologous cell therapy appears significantly higher than that reported in no-option CLTI patients treated with conventional therapy (standard wound care and best practice medical therapy), where major amputation rates were nearly 40% [26].

From a clinical perspective, mechanical revascularization aimed at restoring complete foot perfusion should remain the first-line treatment. Revascularization of the foot arteries (including pedal and plantar angioplasty) is associated with lower major amputation rates compared to PB-MNC therapy (2–12% vs. 10–20%) [24–30]. At the same time, PB-MNCs therapy in no-option CLTI patients results in significantly lower amputation rates compared to the conventional treatment alone (10–20% vs. 30–36%) [7, 24–26].

The risk of major amputation in no-option CLTI patients is comparable to that observed in patients with ischemic DFUs who are not candidates for revascularization. For example, Faglia et al. reported a 34.2% major amputation rate, and the persistence of limb ischemia has been documented to be the main predictor of amputation [8, 9].

A recent review showed that patients treated with autologous cell therapy (ACT) had a significantly lower risk of major amputation compared to standard care alone (OR 0.47), higher wound healing rates (OR 10.1), greater improvements in TcPO₂ and ankle–brachial index (WMD 17.57), and reduced foot pain (WMD − 1.83) over a follow-up of at least 26 weeks [31].

As reported above, while the possibility of limb salvage in no-option CLTI patients is increased using cell-therapy, other concomitant factors are related to the risk of major amputation. In this specific case, ESRD and heel location have already been documented to be two predictors of major amputation due to the associated severity of limb ischaemia in dialysed patients and for the complex anatomical and biological characteristics of the heel, which is per se an ischaemic area because of the anatomical absence of muscle tissue, the risk of bone involvement and for the extreme difficulty to reconstruct after ablative surgery [3, 32–37]. Heel location was found in 26.9% of cases, indicating that this was a particularly challenging population to manage not only for the persistence of limb ischaemia but also for severe aspects of wound characteristics and location. It is also not surprising about the negative predictor role of the presence of multiple ulcers, having been already documented as a risk factor for unsuccessful outcomes in no-option patients managed by cell therapy [37]. 

The least favourable outcome in our study is the relatively high mortality rate, with 27.1% of patients having died at 2 years. Almost all deaths were due to cardiovascular causes, mainly acute ischemic heart disease and heart failure. This finding is likely explained by the high baseline prevalence of chronic ischaemic heart disease (72.9%) and heart failure (33.3%) and confirmed by the multivariate analysis where heart disease (both ischaemic and heart failure) was found to be an independent predictor of 2-year mortality. The mortality rate appears lower than that reported in no-option CLTI patients treated with conventional therapy (approximately 46.7%), but slightly higher than the 20% reported at 2 years by Scatena et al. [26]. This difference may be partly explained by the lower prevalence of cardiovascular disease (60.5%) in their study population [26].

Beyond confirming long-term limb salvage, one of the most interesting findings of the present study is the potential sustained vascular protection over time. Only 6.2% of patients required a new lower-limb revascularization during the 2-year follow-up due to recurrent ischaemia in the treated limb. In all cases, reintervention was related to new stenosis or occlusions in vessels above-the-ankle, occurring in patients with persistent wounds where cell therapy alone was insufficient to ensure adequate perfusion in the wound angiosome.A recent study during 6-month follow-up found that 21.9% of patients required re-admission and 10.9% were readmitted due to a new ischaemic condition [38]. Despite the shorter follow-up, the rate of ischaemic re-admission remains slightly higher than that observed in the cohort of the present study.

Overall, our data indicate that PB-MNC therapy could also give potential benefits in a long-term period when combined with best practice standard of care. It also appears to contribute to sustained foot perfusion, as suggested by the low recurrence rate of limb ischemia, even though these data exclude amputees and deceased patients.

Improved foot perfusion following PB-MNC therapy has been widely reported in the literature [22–26, 39], although in most studies follow-up did not exceed 1 year. The beneficial vascular effects of autologous cell therapy in patients with below-the-ankle disease and small-vessel involvement may be explained by its mechanism of action, which promotes collateral vessel formation and vascular remodelling within the wound angiosome [25, 40, 41].

Even though patients treated with PB-MNCs were reported to have significantly better outcomes overall, it should be rightly reported that those managed by conventional therapy had a higher rate of ESRD and heart failure at baseline assessment, and both of them are independent risk factors for major amputation and mortality respectively [3, 7]. The study seems to document the potential benefits of cell therapy, but it should be highlighted that the concomitant impact of comorbidities and wound characteristics in the management of this high-risk population, being that outcomes are not only related to a specific factor but to several complex variables.

Although studies on PB-MNC therapy are heterogeneous in terms of inclusion criteria, treatment protocols, PAD characteristics, and outcome measures, a reduction in major amputation rates compared to conventional therapy has been consistently reported.

However, comparisons with the literature should be interpreted with caution. It should be highlighted that comparing current data with literature data could mislead some conclusions for different reasons: patient populations differ significantly in clinical characteristics, DFUs severity and comorbidities. In addition, treatment protocols could vary across centres, and outcome definitions are not standardized. Therefore, any outcome reported and authors’ interpretation should be critically appraised.

In conclusion, our study suggests the potential benefits of long-term effects of PB-MNC therapy and suggests also a potential protective role in reducing hospital readmissions due to recurrent limb ischaemia.

The study has several limitations. It is a single-centre study with a relatively small sample size, although comparable to similar studies in this field. The selection criteria, not considering results from excluded patients, may have led to overestimation of favourable outcomes. Even though data are very interesting, due to having a control group, it should be highlighted that patients treated with conventional therapy presented more severe clinical characteristics at the assessment and this pattern influenced the measures of outcome. One of the major limitations is the inclusion of a non-contemporaneous historical control group, which introduces potential temporal confounding that cannot be fully controlled and therefore limits the ability to draw causal inferences. Nonetheless, both the study and control group have been managed by a multidisciplinary team with the same limb salvage protocol.

Patients were managed by an experienced multidisciplinary team in a specialized tertiary care centre, receiving care which includes advanced wound care, vascular interventions, and surgical management. The need and impactful role of the multidisciplinary team in complex diabetic foot disease has already been documented, and these factors may have significantly influenced outcomes and could limit the generalizability of the results to other clinical settings.

Further studies are needed to better define patient selection criteria (clinical and vascular), optimal timing of treatment, repeatability of the procedure, and its role in combination with subsequent surgical strategies.

## Conclusions

PB-MNCs therapy should be considered a current therapeutic option for patients with ischaemic DFUs and no-option CLTI. Further studies, mainly RCTs, are required to reinforce its potential benefit and to better clarify its role and indications. Autologous cell therapy should also be considered a potentially useful treatment in these ischaemic patients who are often considered unsalvageable due to the failure of mechanical revascularization. When patients with no-option CLTI present with several comorbidities and a considerable risk of death from cardiovascular causes, close screening of heart complications should always be mandatory.
